
JOURNAL INFORMATION
==============================
NLM Title Abbreviation: Innov Aging
Journal ID: Innov Aging
NLM Journal ID: 101703706
Journal ID: innovateage

Innovation in Aging

EISSN: 2399-5300
Publisher: Oxford University Press

ARTICLE INFORMATION
==============================
PMCID: PMC12759667
DOI: 10.1093/geroni/igaf122.2058
Article ID: igaf122.2058
Article version: 1
Subjects: Session 4473 (Biological Sciences Invited Symposium), AcademicSubjects/SOC02600

PROTEOMIC ANALYSIS OF THE SENESCENCE-ASSOCIATED SECRETORY PHENOTYPE: GDF-15, IGFBP-2, AND CYSTATIN-C ARE ASSOCIATED WITH MULTIPLE AGING TRAITS

Schilling Birgit Champalimaud Foundation, Lisbon, Portugal

Evans Daniel California Pacific Medical Center Research Institute, San Francisco, California, United States

Young Danielle Nestlé Research, Lausanne, Vaud, Switzerland

Watson Mark University of Minnesota, Minneapolis, Minnesota, United States

Tanaka Toshiko University of Florida, Gainesville, Florida, United States

Basisty Nathan National Institutes of Health, Gaithersburg, Maryland, United States

Bandinelli Stefania University of Florida, Gainesville, Florida, United States

Duncan Francesca Nestlé Research, Lausanne, Switzerland

Publication date: 2025 Dec
Electronic publication date: 2025 Dec 31
Volume: 9
Issue: Suppl 2
Issue title: Program Abstracts from the GSA 2025 Annual Scientific Meeting, “Innovative Horizons in Gerontology”
Electronic Location ID: igaf122.2058
Copyright: © The Author(s) 2025. Published by Oxford University Press on behalf of the Gerontological Society of America.
Copyright year: 2025
License: This is an Open Access article distributed under the terms of the Creative Commons Attribution License (https://creativecommons.org/licenses/by/4.0/), which permits unrestricted reuse, distribution, and reproduction in any medium, provided the original work is properly cited.
License URL: https://creativecommons.org/licenses/by/4.0/

Page count: 1

==============================
Abstract

